# Supplementary material for: Formulation development and comparability studies with an aluminum-salt adjuvanted SARS-CoV-2 spike ferritin nanoparticle vaccine antigen produced from two different cell lines
Source: Vaccine. Author manuscript; Available in PMC 2024 Jun 17. (PMC11181998; doi:10.1016/j.vaccine.2023.08.037)
Supplement: 3 [file NIHMS2001717-supplement-3.pdf]

Glycan	Average Relative Fluorescence Peak Area Abundance ± 1SD (%)	Average Relative Ion Abundance ± 1SD (%)	Structure
H3N4F1	21.4 ± 0.5	24.5 ± 0.1	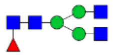
H5N2	13.1 ± 0.0	12.6 ± 0.1	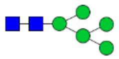
H3N5F1	9.6 ± 0.3	10.6 ± 0.0	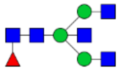
H4N4F1	6.4 ± 0.1	7.2 ± 0.1	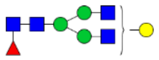
H3N3F1	5.6 ± 0.1	5.6 ± 0.0	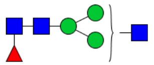
H4N4F1S1	10.2 ± 0.1* (elutes at same time as H5N4F1)	5.4 ± 0.0	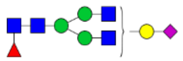
H4N4F1	6.4 ± 0.1	3.5 ± 0.0	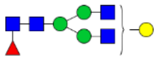
H4N5F1	2.0 ± 0.1	2.8 ± 0.0	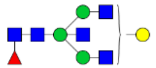
H5N4F1	10.2 ± 0.1* (elutes at same time as H4N4F1S1)	2.8 ± 0.0	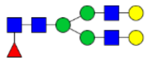
H7N2	4.4 ± 0.1	2.6 ± 0.0	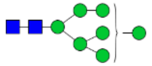

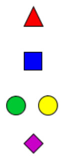

Fuc
HexNAc
Hex
NeuAc
