# Supplementary material for: Formulation development and comparability studies with an aluminum-salt adjuvanted SARS-CoV-2 spike ferritin nanoparticle vaccine antigen produced from two different cell lines
Source: Vaccine. Author manuscript; Available in PMC 2024 Jun 17. (PMC11181998; doi:10.1016/j.vaccine.2023.08.037)
Supplement: 1 [file NIHMS2001717-supplement-1.pdf]

	CHO	Expi293
Overall Sequence Coverage (not including signal peptide)	94%	92%
Sequence Coverage of Spike Portion	93%	91%
Sequence Coverage of Ferritin Portion	98%	98%

CHO DCFHP
Expi293 DCFHP
Ferritin

```
        10         20         30         40         50         60         70         80         90        100        110        120
MFVFLVLLPL VSSQCVNLTT RTQLPPAYTN SFTRGVYYPD KVFRSSVLHS TQDLFLPFFS NVTWFHAIHV SGTNGTKRFD NPVLPFNDGV YFASTEKSNI IRGWIFGTTL DSKTQSLLIV

       130        140        150        160        170        180        190        200        210        220        230        240
NNATNVVIKV CEFQFCNDPF LGVYYHKNNK SWMESEFRVY SSANNCTFEY VSQPFLMDLE GKQGNFKNLR EFVFKNIDGY FKIYSKHTPI NLVRDLPQGF SALEPLVDLP IGINITRFQT

       250        260        270        280        290        300        310        320        330        340        350        360
LLALHRSYLT PGDSSSGWTA GAAAYYVGYL QPRTFLLKYN ENGTITDAVD CALDPLSETK CTLKSFTVEK GIYQTSNFRV QPTESIVRFP NITNLCPFGE VFNATRFASV YAWNRKRISN

       370        380        390        400        410        420        430        440        450        460        470        480
CVADYSVLYN SASFSTFKCY GVSPTKLNDL CFTNVYADSF VIRGDEVRQI APGQTGKIAD YNYKLPDDFT GCVIAWNSNN LDSKVGGNYN YLYRLFRKSN LKPFERDIST EIYQAGSTPC

       490        500        510        520        530        540        550        560        570        580        590        600
NGVEGFNCYF PLQSYGFQPT NGVGYQPYRV VVLSFELLHA PATVCGPKKS TNLVKNKCVN FNFNGLTGTG VLTESNKKFL PFQQFGRDIA DTTDAVRDPQ TLEILDITPC SFGGVSVITP

       610        620        630        640        650        660        670        680        690        700        710        720
GTNTSNQVAV LYQDVNCTEV PVAIHADQLT PTWRVYSTGS NVFQTRAGCL IGAEHVNNSY ECDIPIGAGI CASYQTQTNS PGSASSVASQ SIIAYTMSLG AENSVAYSNN SIAIPTNFTI

       730        740        750        760        770        780        790        800        810        820        830        840
SVTTEILPVS MTKTSVDCTM YICGDSTECS NLLLQYGSFC TQLNRALTGI AVEQDKNTQE VFAQVKQIYK TPPIKDFGGF NFSQILPDPS KPSKRSPIED LLFNKVTLAD AGFIKQYGDC

       850        860        870        880        890        900        910        920        930        940        950        960
LGDIAARDLI CAQKFNGLTV LPPLLTDEMI AQYTSALLAG TITSGWTFGA GPALQIPFPM QMAYRFNGIG VTQNVLYENQ KLIANQFNSA IGKIQDSLSS TPSALGKLQD VVNQNAQALN

       970        980        990       1000       1010       1020       1030       1040       1050       1060       1070       1080
TLVKQLSSNF GAISSVLNDI LSRLDPPEAE VQIDRLITGR LQSLQTYVTQ QLIRAAEIRA SANLAATKMS ECVLGQSKRV DFCGKGYHLM SFPQSAPHGV VFLHVTYVPA QEKNFTTAPA

      1090       1100       1110       1120       1130       1140       1150       1160       1170       1180       1190       1200
ICHDGKAHFP REGVFVSNGT HWFVTQRNFY EPQIITTDNT FVSGNCDVVI GIVNNTVYDP LQPELDSGGD IIKLLNEQVN KEMQSSNLYM SMSSWCYTHS LDGAGLFLFD HAAEEYEHAK

      1210       1220       1230       1240       1250       1260       1270       1280       1290       1300       1310
KLIIFLNENN VPVQLTSISA PEHKFEGLTQ IFQKAYEHEQ HISESINNIV DHAIKSKDHA TFNFLQWYVA EQHEEEVLFN DILDKIELIG NENHGLYLAD QYVKGIAKSR KS
```
