# Supplementary material for: Formulation development and comparability studies with an aluminum-salt adjuvanted SARS-CoV-2 spike ferritin nanoparticle vaccine antigen produced from two different cell lines
Source: Vaccine. Author manuscript; Available in PMC 2024 Jun 17. (PMC11181998; doi:10.1016/j.vaccine.2023.08.037)
Supplement: 5 [file NIHMS2001717-supplement-5.pdf]

| Formulation | Final DCHFP concentration (mcg/mL) | Antigen amount (per 0.1 mL) (mcg) | Aluminum concentration (mg/mL) | Aluminum amount (per 0.1 mL) (mcg) | IM route injection volume (mL) | % Antigen bound to AH |
|---|---|---|---|---|---|---|
| AH | 100 | 10 | 1.5 | 150 | 0.1 | 100% |
| AH+20mM sodium phosphate | 100 | 10 | 1.5 | 150 | 0.1 | ~40% |
| AH+200mM sodium phosphate | 100 | 10 | 1.5 | 150 | 0.1 | ~10% |
| No adjuvant | 100 | 10 | 0 | 0 | 0.1 | N/A |
