# Supplementary material for: Formulation development and comparability studies with an aluminum-salt adjuvanted SARS-CoV-2 spike ferritin nanoparticle vaccine antigen produced from two different cell lines
Source: Vaccine. Author manuscript; Available in PMC 2024 Jun 17. (PMC11181998; doi:10.1016/j.vaccine.2023.08.037)

Glycan	Average Relative Fluorescence Peak Area Abundance ± 1SD (%)	Average Relative Ion Abundance ± 1SD (%)	Structure
H5N4F1	13.8 ± 0.3	14.1 ± 0.1	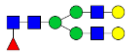
H5N2	10.7 ± 0.4	13.8 ± 0.2	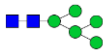
H5N4F1S1	10.9 ± 0.2	10.9 ± 0.2	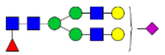
H4N4F1	4.4 ± 0.0	4.2 ± 0.0	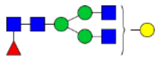
H6N2	3.0 ± 0.1	4.2 ± 0.0	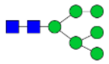
H6N5F1	5.1 ± 0.1	4.2 ± 0.1	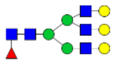
H7N2	3.6 ± 0.1	4.1 ± 0.0	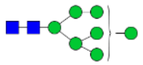
H5N4	3.4 ± 0.0	3.8 ± 0.1	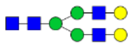
H6N5F1S1	4.8 ± 0.1	3.8 ± 0.1	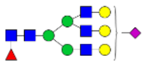
H5N4F1S2	4.5 ± 0.1	3.3 ± 0.1	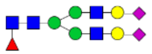

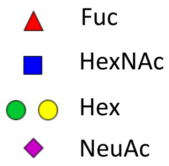

Supplement: 4 [file NIHMS2001717-supplement-4.pdf]
